# Source Identification and Apportionment of Trace Elements in Soils in the Yangtze River Delta, China

**DOI:** 10.3390/ijerph15061240

**Published:** 2018-06-12

**Authors:** Shuai Shao, Bifeng Hu, Zhiyi Fu, Jiayu Wang, Ge Lou, Yue Zhou, Bin Jin, Yan Li, Zhou Shi

**Affiliations:** 1 Institute of Applied Remote Sensing and Information Technology, Zhejiang University, Hangzhou 310058, China; sshuai@zju.edu.cn (S.S.); fzy513432821@gmail.com (Z.F.); wangjiayu_zju@163.com (J.W.); lllouxbai@outlook.com (G.L.); yuezhou@zju.edu.cn (Y.Z.); shizhou@zju.edu.cn (Z.S.); 2 Science du Sol, INRA, 45075 Orléans, France; bifeng.hu@inra.fr; 3 Unité InfoSol, INRA, US 1106, 45075 Orléans, France; 4 Sciences de la Terre et de lthe’Univers, Orléans University, 45067 Orleans, France; 5 Ningbo Agricultural Food Safety Management Station, Ningbo 315000, China; dd923068626@163.com; 6 Institute of Land Science and Property, School of Public Affairs, Zhejiang University, Hangzhou 310058, China

**Keywords:** trace elements, source identification and apportionment, principal component analysis (PCA), finite mixture distribution model (FMDM), Yangtze River Delta

## Abstract

Trace elements pollution has attracted a lot of attention worldwide. However, it is difficult to identify and apportion the sources of multiple element pollutants over large areas because of the considerable spatial complexity and variability in the distribution of trace elements in soil. In this study, we collected total of 2051 topsoil (0–20 cm) samples, and analyzed the general pollution status of soils from the Yangtze River Delta, Southeast China. We applied principal component analysis (PCA), a finite mixture distribution model (FMDM), and geostatistical tools to identify and quantitatively apportion the sources of seven kinds of trace elements (chromium (Cr), cadmium (Cd), mercury (Hg), copper (Cu), zinc (Zn), nickel (Ni), and arsenic (As)) in soil. The PCA results indicated that the trace elements in soil in the study area were mainly from natural, multi-pollutant and industrial sources. The FMDM also fitted three sub log-normal distributions. The results from the two models were quite similar: Cr, As, and Ni were mainly from natural sources caused by parent material weathering; Cd, Cu, and Zu were mainly from mixed sources, with a considerable portion from anthropogenic activities such as traffic pollutants, domestic garbage, and agricultural inputs, and Hg was mainly from industrial wastes and pollutants.

## 1. Introduction

Trace elements pollution has attracted a lot of attention around the world [1,2,3,4,5]. There is much concern about trace elements-contaminated soils because of their high toxicity and resistance to degradation [6]. Trace elements pose high risks to health and ecosystem function when introduced to the human body and ecosystems via food chains, respectively [7,8,9]. The sources of soil trace elements are generally natural or anthropogenic [10,11]. Formerly, trace elements were mainly introduced to soils through weathering of parent materials but with rapid increases in urban, industrial, and agricultural activities, such as electronic plating, fossil fuel combustion, and agricultural chemical fertilizer abuse, in recent years, the high trace element concentrations in soil now mainly reflect human activities [3,12]. The geographical distribution of trace elements in soil is complex [7]. In order to repair polluted soils and prevent further soil pollution, we need to qualitatively and quantitatively identify and apportion the sources of trace elements. This information can help decision-makers understand how trace elements vary spatially and why their concentrations fluctuate erratically which is a necessary prerequisite to mitigating and preventing soil pollution [13].

Identifying and apportioning the sources of soil trace elements dates back to the 1970s [14]. Various methods, including chemical mass balance (CMB) [15], molecular markers (MM) [16], isotope tracing (IT) [17], UNMIX models [18], factor analysis (FA) [19], positive definite matrix factor analysis (PMF) [20], and multiple linear regression (APCS-MLR) [21], are currently used to identify the sources of trace elements in soil. Applications of such models are generally limited by their rigorous demands for preliminary information and numerous datasets comprising high resolution data, for example, about the number of pollution sources, properties of soils from the source areas, and the comparative stability of source transport processes [22,23].

Principle component analysis (PCA) is a widely-used multi-statistical analysis method whose purpose is to switch multidimensional data into a couple of relevant variables that simultaneously maintain the information involved in the original variables and diminish the dimensions of the data [24,25,26]. While finite mixture distribution model (FMDM) is a mathematical approach for statistical modeling of massive random data sets [27]. It can be applied to identify whether the trace elements from soil samples originate from a natural background or anthropogenic background, without knowing the general contaminated condition of the full areal extent of the study area [28,29]. But, to date, few studies have used and compared different models to identify the sources of trace elements. To fill this gap, in our study, we compared the abilities of PCA and FMDM to identify and apportion the sources of trace elements in soils in the Yangtze River Delta. The main objectives of this study were to (1) describe and estimate the concentrations and distribution of trace elements in soil and assess the pollution status of the Yangtze River Delta; (2) apply PCA and FMDM to determine the sources of seven trace elements over a large area; and (3) to confirm and apportion the main sources of trace elements in soil.

## 2. Materials and Methods

### 2.1. Study Area and Sampling

**Figure 1:**
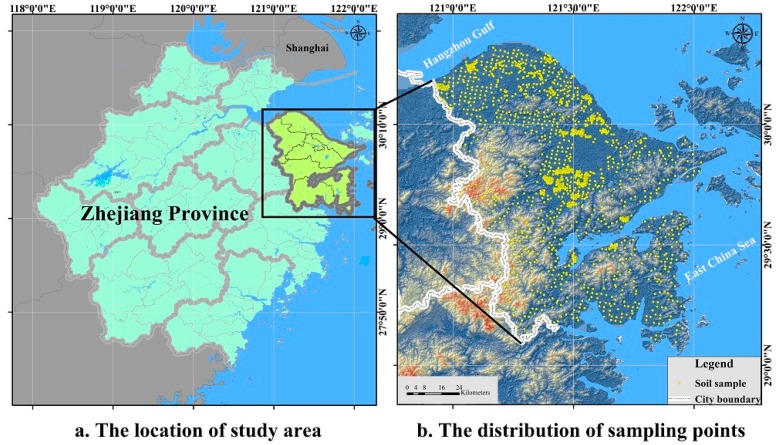
Maps showing the location of the study area (**a**) and the distribution of sampling points (**b**).

The study area is a crucial port in the Yangtze River Delta, China (Figure 1). The area is dominated by plains and low hills. It has an average elevation of about 4.2 m and the elevation generally decreases from the southwest to the northeast. A total of 2051 topsoil (0–20 cm) samples were collected from the study area, which was first divided into strata according to land use type, and systematic grid sampling was applied. At some of the grid nodes, grid sampling was augmented by sampling nearby areas (Figure 1). We collected a soil sample at an intersection point and combined it with five subsamples collected from five locations within 5 m. The coordinates of the sampling locations were recorded with a differential global positioning system. 

We air-dried the soil samples in the lab in ambient air for several days, then passed them through a 2-mm nylon sieve and save for further analysis of soil properties [30]. Soil pH was measured by the Glass Electrode method with a soil/solution of 1:2.5 (*m*/*v*); The total concentration of Cr, As, Cu, Cu, Zn, and Ni of soil samples were all digested with the acid HCl-HNO_3_-HClO_4_ and total Cd was acid-digested with HF-HNO_3_-HClO_4_; As for total Hg, it was digested by a double channel Atomic Fluorescence Spectrometer with the digestion of HNO3-HCl bathing in the hot water. We use reagent blanks and standard reference in the whole analysis procedure for quality assurance and quality control, and the recovery ranges of the trace elements were from 90 to 110% [31].

### 2.2. Auxiliary Variables Data

The study area is known for its industrial prosperity, and its total industrial output value accounted for 45% of its whole economic output value in 2016. We obtained information of 48,206 industrial enterprises in the study area from Baidu Map POI (Point of Interest) data that listed all the industrial enterprises in 2016. We removed data for irrelevant non-industrial enterprises, and then classified the industrial enterprises into four categories: textiles, metal products, chemical products, and other industries. We obtained information about the soil parent material in the study area from a 1:20,000 soil map of Zhejiang Province (1990) [32] and the soil types from a 1:50,000 Map of Chinese Soil (1990) published by the National Soil Survey of China, respectively.

### 2.3. Single Pollution Index (SPI)

We used the single pollution index (SPI) to assess the pollution degree of trace elements contents in soil. The SPI is calculated as follows:

(1) $$P_{i}=\frac{C_{i}}{S_{i}}$$

where *P_i_* is the SPI of trace element *i* in soil, *C_i_* is the test value of trace element *i*, and *S_i_* is the regulation value in China [33]. When *P_i_* is less than or equal to 1, the content of the soil trace element *i* is within a safe range, while when *P_i_* is within the range of 1 to 2, the content of the soil trace element *i* slightly exceeds the standard value; when *P_i_* is within the range of 2 to 3, soils could be moderately contaminated by trace element *i*; and when *P_i_* is greater than 3, soils could be severely contaminated by trace element *i*.

### 2.4. Ordinary Kriging (OK)

We used the widely-used ordinary kriging (OK) model to show the spatial variations of each soil trace element across the study area. The equation for this model is as follows [34,35]:

(2) $$Z^{*}\left(x_{0}\right)=\overset{n}{\underset{i=1}{\sum }}\varphi_{i}Z\left(x_{i}\right)$$

where Z*(*x*_0_) is the linear prediction value while *Z*(*x_i_*) is the observed value. *n* and *i* denote the quantities of observed and predicted samples, respectively, and *φ_i_* is the optimal weight value that results in an unbiased prediction with the minimum variance. 

### 2.5. Principle Component Analysis (PCA)

We distinguished different groups of trace elements with PCA. This tool uses an orthogonal transformation to convert a set of observations of possibly correlated variables into a set of values of linearly uncorrelated variables. We then used non-transformed data to calculate the correlation matrix, performed an orthogonal rotation based on the Kaiser Standard, and extracted factors with eigenvalues greater than 1 after a maximum of 25 iterations [36].

### 2.6. Finite Mixture Distribution Model (FMDM)

For a random variable *x*, if a mixture distribution consists of m components and the distribution of the *i*th individual component is determined by a specific probability density function (pdf) *f_i_*(*x*), then the general pdf *f*(*x*) for the mixture distribution can be expressed as [27,37]:

(3) $$f\left(x\right)=\overset{m}{\underset{i=1}{\sum }}\pi_{i}f_{i}\left(x\right)=\pi_{1}f_{1}\left(x\right)+\ldots +\pi_{m}f_{m}\left(x\right)$$

(4) $$\overset{m}{\underset{i=1}{\sum }}\pi_{i}=1\left(0\leq \pi_{i}\leq 1\right)$$

where *π_i_* denotes the mixed weights of every sub-distribution.

Many natural processes follow a normal distribution or a log-normal distribution. Here, we used a log-normal distribution as the pdf to describe the trace element content of soils from different sources [38], as follows:

(5) $$f_{i}\left(x\;│\mu_{m},\sigma_{m}\right)=\frac{1}{\sqrt{2\pi }\sigma_{m}x}e^{-\frac{(lnx-\mu_{m}{)}^{2}}{2\sigma_{m}{}^{2}}},x>0$$

where *µ_m_* and *σ_m_* represent the mean and standard deviation of every sub-distribution, which can be obtained using an expectation maximization algorithm [39]. We then used the Chi-square goodness-of-fit test to test the null hypothesis *H*_0_ to confirm that the assumed model was consistent with the observed distribution. The cut-off value between the *i*th and (*i* + 1) components can be calculated after the above parameters have been determined, as follows:

(6) $$\pi_{i}\int_{a_{0}}^{+\infty }f_{i}\left(x\right)dx=\pi_{i+1}\int_{-\infty }^{a_{0}}f_{i+1}(x)dx$$

### 2.7. Data Analysis

In this study, we did all the statistical analyses with Microsoft Excel 2016 (Office 2016, Redmond, WA, USA). We used the geostatistical analyst tool in ArcGIS 10.2 (ESRI, ArcGIS10.2, Redlands, CA, USA) for the kriging interpolation. Principal component analysis of the seven soil trace elements was carried out with the psych package of R3.4.2 [40] and the FMDM was done in R3.4.2 using Mclust package [41].

## 3. Results and Discussion

### 3.1. Summary Statistical Analysis of Soil Trace Elements

The average concentrations of Cr, Cd, Hg, As, Cu, Zn, and Ni in the study area were 67.72, 0.197, 0.288, 6.58, 34.77, 110.67, and 29.22 mg/kg, respectively (Table 1). Apart from Cr, the average values of the trace elements were higher than the background values; the averages of Hg, Cd, Zn, and Cu were much higher than the background values which suggest that these trace elements may have been affected by human activities [26]. 

The coefficient of variation (CV) reflects the degree of dispersion of the sample data [42]. The CV values of the soil trace elements in the study area were ranked as follows: Hg (104.55%) > Ni (56.96%) > Cd (50.68%) > Cu (47.99%) > Cr (43.41%) > As (40.33%) > Zn (33.0%). The uneven distribution of Hg reflects anthropogenic impacts, and the other trace elements also showed different degrees of variability.

### 3.2. Analysis of Auxiliary Variable Data

**Figure 2:**
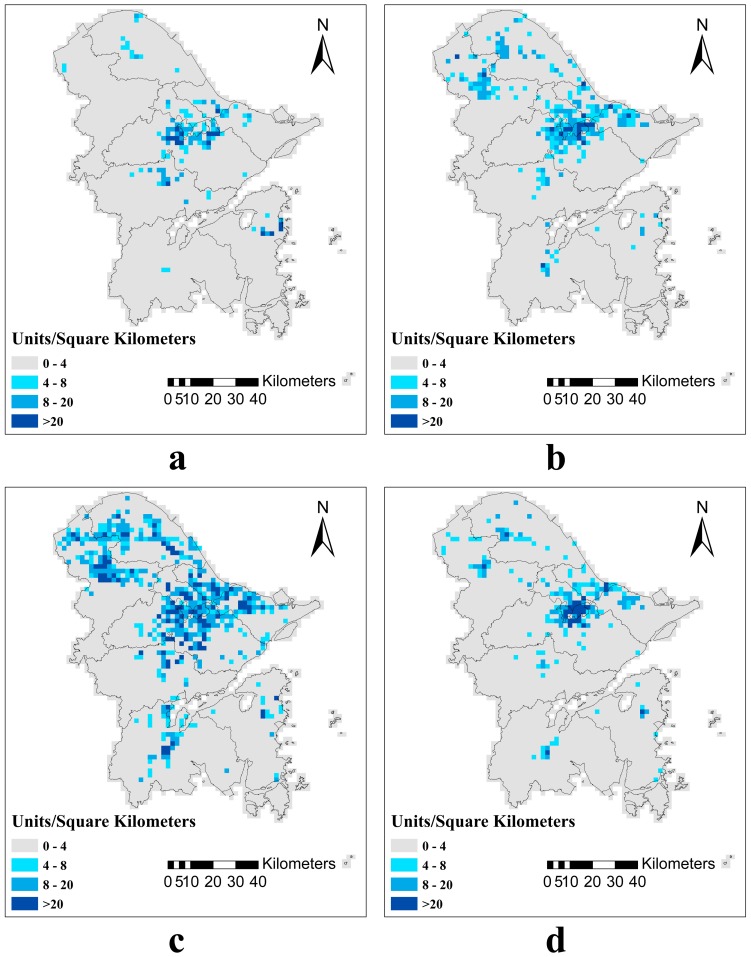
The density maps of (**a**) textile enterprises; (**b**) chemical product enterprises; (**c**) metal product enterprises; and (**d**) other enterprises in the study area (Units/km^2^).

As shown in previous studies, industrial pollution is a major source of trace element pollution in the soil [44,45,46]. Trace elements in industrial waste water, waste gas, and waste residues enter the soil via sewage irrigation, garbage dumping, and atmospheric deposition, resulting in high trace element concentrations in the soil [46,47,48]. Analysis of the density of all the industrial enterprises (Figure 2) in the study area shows that the density was highest for the metal product enterprises, followed by chemical product enterprises, then textiles, and was lowest for other enterprises, and that most enterprises were clustered around the urban city areas.

As Table 2 indicates, the Cr and Ni concentrations increased as the quantity of textile enterprises increased, reflecting the quantitative impacts of the textile industry on these two trace elements. Similarly, chemical product enterprises influenced the concentrations of Hg, Cu, Zn, and Ni; metal product enterprises influenced the concentrations of Cr, Hg, Cu, and Zn, and other enterprises influenced the Ni, Hg, and Cu concentrations. In general, as the total number of all enterprises increased, the contents of all seven trace elements increased dramatically, which shows that industrial activities were a major contributor to trace elements in soils.

**Figure 3:**
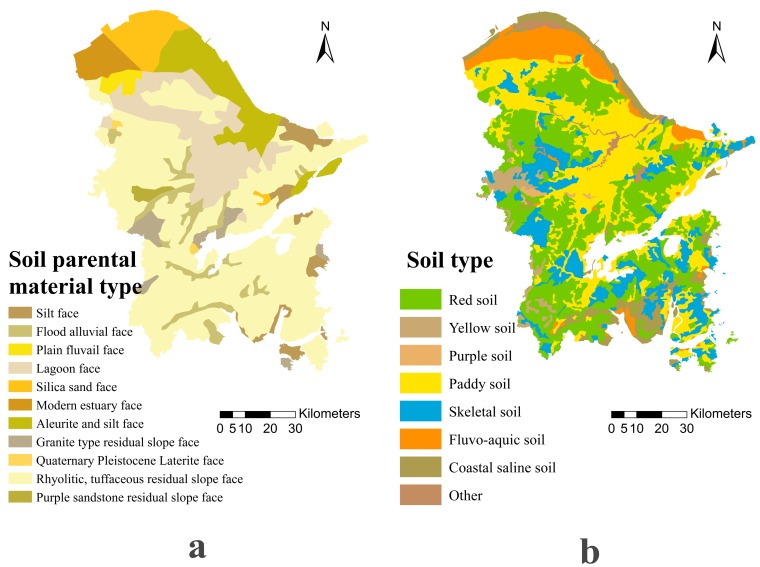
The distribution of the (**a**) soil parent materials and (**b**) soil types in the study area.

Rhyolitic, tuffaceous residual slope faces dominated the parent materials (Table 3 and Figure 3a) in the study area. Lakes and marshes extended over large parts of the north-central part of the study area, while coastal sedimentary parent materials covered large areas of the northern and southern parts. The main soil types (Table 4 and Figure 3b) were divided into paddy, fluvo-aquic, red, coastal saline, skeletal, yellow, and purple soils. Paddy soils and red soils dominated the study area; red soils were distributed across the entire study area and paddy soils were mainly distributed over most of the central and northern parts. Fluvo-aquic and coastal saline soils were mostly distributed in the northern coastal areas, and skeletal soils were distributed in the central and southern regions.

### 3.3. Assessment of Trace Element Pollution

The SPI values showed that, apart from Hg, the soils were only slightly polluted by trace elements. The soils from more than 90% of the sampling points had *P_i_* values less than or equal to 1 (Table 5), which indicates that the other 6 trace elements did not pose much risk to the environment or ecology. There are obvious signs of Hg contamination in the study area and, with 18.97%, 6.05%, and 5.70% of the soils lightly, moderately, and severely polluted, respectively, around 30% of the soils from the study area were adversely impacted by Hg.

### 3.4. Spatial Distribution of Soil Trace Elements

**Figure 4:**
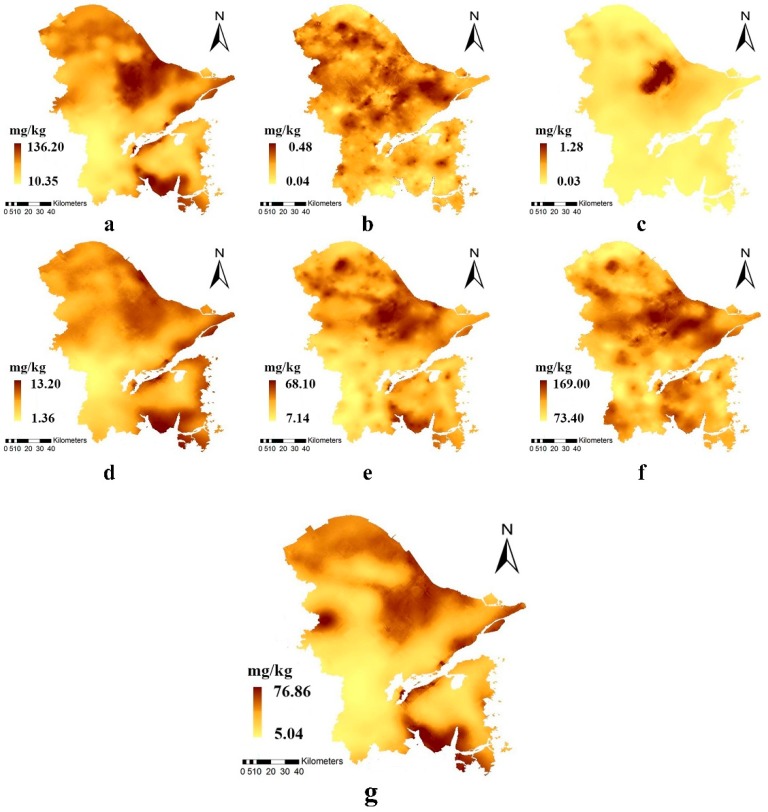
Spatial distributions of (**a**) Cr; (**b**) Cd; (**c**) Hg; (**d**) As; (**e**) Cu; (**f**) Zn; and (**g**) Ni.

The general spatial distribution of trace elements in soils in the study area is presented in Figure 4. Cr, As, and Ni concentrations were highest in the central urban areas and the coastal areas (Figure 4a,d,g). It is revealed by Table 3 that the main sources of Cr, As, and Ni were aleurite and silt face (with silt face as the dominant source in the coastal areas), and the fact that the concentrations of these trace elements were highest in the urban centers indicates that they were closely related to anthropogenic activities. As shown in Figure 4b,f, areas with high Cd and Zn concentrations were relatively scattered and were mainly concentrated in the northern and central urban areas. The chemical industries probably influenced the Cd and Zn concentrations (Table 2). Concentrations of Hg and Cu were highest in the central parts of the urban areas (Figure 4c,f), and close to the metal and textile industries (Table 2).

### 3.5. Source Identification Based on PCA

**Figure 5:**
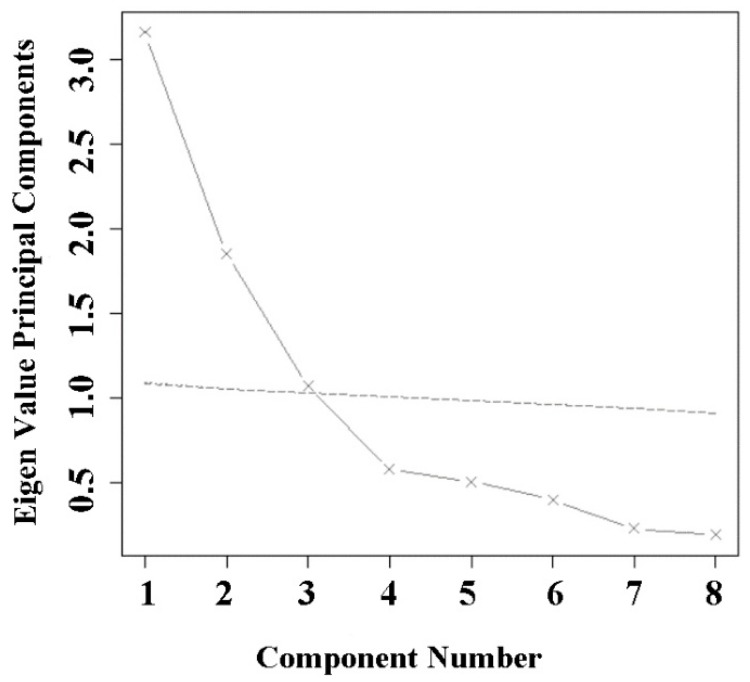
Cattell’s scree plot (a parallel analysis of 100 simulations).

The Cattell’s scree plot (Figure 5) shows that the seven trace elements fell into three main components, while the PCA (Table 6) results show that the cumulative contribution rate of the three main components was up to 70.0%. The first principal component (PC1) contained three trace elements, Cr, As, and Ni; the second principal component (PC2) contained Cd, Cu, and Zn, and the third component (PC3) represented Hg. PC1, PC2, and PC3 accounted for 30%, 25%, and 15%, respectively.

**Figure 6:**
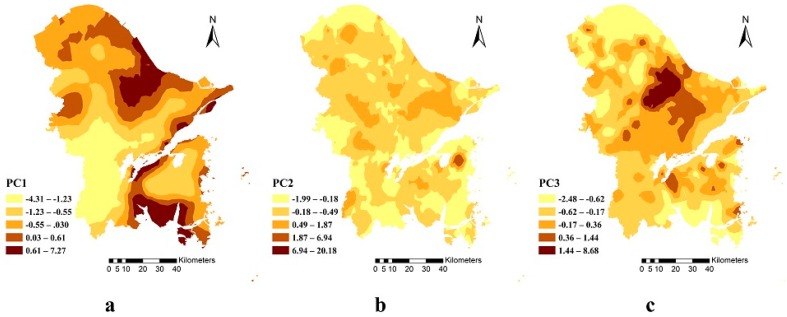
OK interpolation maps of (**a**) PC1; (**b**) PC2; (**c**) PC3.

The contribution of PC1 was up to 30% (Table 6), and had factor loadings of 0.88, 0.73, and 0.87 for Cr, As, and Ni, respectively. This suggests Cr, As, and Ni had the same pollution source. The OK spatial interpolation map (Figure 6a) shows that PC1 gradually decreased from the coast to the inland area, where the high-value areas were mainly confined to the city centers, the northeast, and the southern coastal areas. This trend greatly matches previous spatial distribution analysis, so these trace elements were mainly from natural sources.

The contribution of PC2 was 25% (Table 6); Cd, Cu, and Zn had factor loadings of 0.81, 0.72, and 0.82, respectively, which suggest a common pollution source. Alternatively, because Zn and Cu contributed as much as 0.45 and 0.28 to the first principal component, Zn and Cu may have shared the sources for PC1. The areas with high values for PC2 (Figure 6b) were scattered across the study area, with clusters in the northwestern, southern, and central urban parts of the study area. This suggests that PC2 represented mixed pollutant sources, such as traffic exhaust gases, domestic garbage, and agricultural inputs. 

The contribution rate from PC3 was 15%, and the factor loading of Hg was 0.67. The OK interpolation map of PC3 (Figure 6c) shows that the areas with high values were mainly distributed in the central parts of the urban areas, which perfectly matches the spatial distribution map. This shows that industrial pollution was the main source of Hg.

### 3.6. Source Identification Based on FMDM

In general, the FMDM results showed that Cr, As, Hg, and Ni conformed to the log-normal mixture distribution; Cd, Cu, and Zn conformed to the log-normal distribution, and the seven trace elements passed the significance level test (*p* > 0.05).

**Figure 7:**
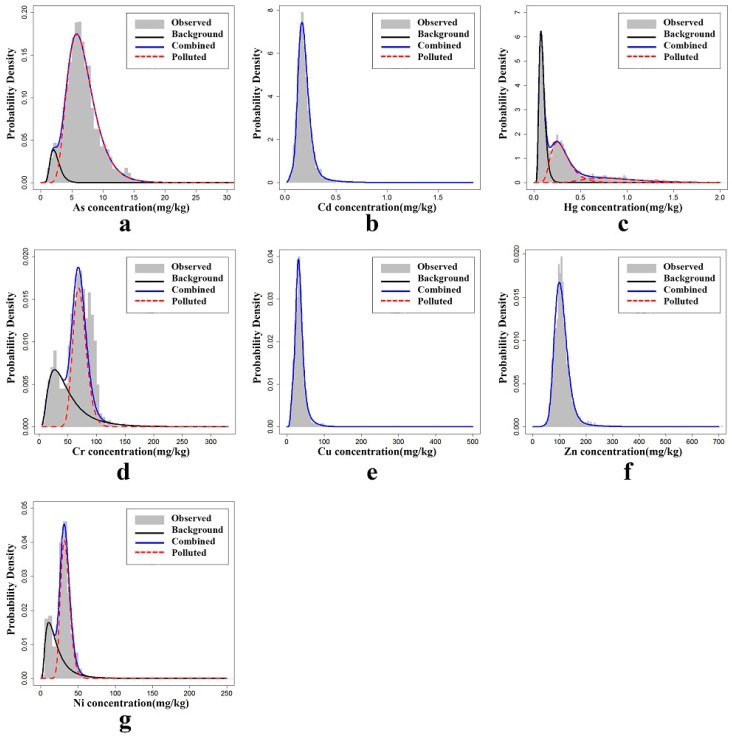
FMDM fit for (**a**) Cr; (**b**) Cd; (**c**) Hg; (**d**) As; (**e**) Cu; (**f**) Zn; and (**g**) Ni.

Cd, Cu, and Zn fitted the single log-normal distribution model as shown in Figure 7b,e,f. The FMDM was not able to clearly identify whether these trace elements had natural or anthropogenic sources, because their sources did not significantly differ throughout the entire study area; the modelled average concentrations were higher than the soil background values, which suggests unnatural sources. So, these three trace elements may have derived in parts from human activities that generate multiple pollutants, including urban development, population increases, domestic garbage, traffic pollution, and agricultural inputs. 

The concentrations of Cr, As, and Ni were all consistent with the double log-normal distribution model (Figure 7a,d,g), and should fall within the natural and anthropogenic distributions in theory. As shown in Table 7, the means of Cr, As, and Ni from the natural distribution were lower than the soil background values (Table 7). The means of the anthropogenic distributions exceeded the soil background values, but were still within the ranges of the background values (Table 1); the FMDM therefore identified two sub-distributions as a low-value natural distribution and a high-value natural and anthropogenic mixed distribution. This suggests that Cr, As, and Ni may have been influenced by anthropogenic activities, but their impacts were not much more than the impacts from a high-value natural background. In such cases, the effects of these trace elements from anthropogenic sources would not be apparent.

The Hg concentrations fit the triple log-normal distribution mode, as shown in Table 7 and Figure 7c. A natural distribution and two anthropogenically-influenced distributions were identified. The modelled mean of natural distribution was below the soil background value, and the modelled means of both anthropogenically-influenced distributions significantly exceeded the soil background value and its maximum range (Table 1), showing that anthropogenic industrial activities were a major influence on the content of Hg in soils in the study area.

## 4. Conclusions

Source identification and apportionment of soil trace elements have always been important prerequisites for treating trace element-contaminated soils and preventing further contamination. In this study, we quantitatively identified the sources of seven trace elements in the Yangtze River Delta using PCA and FMDM. Our results showed that Cr, As, and Ni in soil were mainly from natural sources through weathering of parent materials; Cd, Cu, and Zn were mainly from multiple mixed pollution sources, such as traffic pollutants, domestic garbage, and agricultural inputs, and Hg was mainly from industrial waste and discharge. Our study can efficiently provide valuable information for policy makers and administrators on trace elements contamination. 

## Figures and Tables

**Table 1:** Summary statistics for trace elements in topsoil (mg/kg) [43].

Element	Cr	Cd	Hg	As	Cu	Zn	Ni
Mean	67.72	0.197	0.288	6.58	34.77	110.67	29.22
Median	69.7	0.18	0.19	6.26	33	106	29.8
SD	29.4	0.099	0.301	2.65	16.69	36.52	16.64
CV	43.41%	50.68%	104.55%	40.33%	47.99%	33.00%	56.96%
Range	6.04–326.0	0.03–1.84	0.02–2.26	0.87–19.2	4.28–315.0	34.30–714.0	2.89–293.0
Background Value	68.7	0.157	0.11	6.23	29.2	89.9	26.3
Background Interval	43.0–94.4	0.101–0.213	0.048–0.231	3.94–8.52	9.7–48.6	51.6–128.2	20.6–32.1

SD: standard deviation; CV: coefficient of variation.

**Table 2:** Average trace elements from different numbers of the different enterprises (mg/kg).

Category	Enterprise Quantity	Sample Quantity	Cr	Cd	Hg	As	Cu	Zn	Ni
Textile Industry	0–4	1692	65.40 *	0.197	0.256	6.58	33.80	108.30	28.59 *
	4–8	144	76.94 *	0.203	0.407	6.57	39.18	124.22	31.25 *
	8–20	161	78.03 *	0.193	0.385	6.39	37.97	116.75	32.21 *
	>20	54	85.01 *	0.200	0.695	7.46	43.92	130.57	34.52 *
Chemical materials	0–4	1241	63.10	0.194	0.213 *	6.42	32.04 *	105.64 *	27.87 *
	4–8	298	70.35	0.206	0.336 *	6.79	36.94 *	115.59 *	30.16 *
	8–20	397	76.33	0.195	0.416 *	6.78	38.61 *	116.72 *	31.48 *
	>20	115	81.00	0.219	0.536 *	7.12	45.39 *	131.29 *	33.48 *
Metal products industry	0–4	817	60.21 *	0.188	0.174 *	6.50	31.14 *	104.03 *	27.02 *
	4–8	286	67.84 *	0.204	0.275 *	6.49	32.73 *	107.25 *	28.61 *
	8–20	466	71.59 *	0.198	0.355 *	6.70	36.58 *	111.96 *	31.15 *
	>20	482	76.63 *	0.208	0.426 *	6.67	40.39 *	122.69 *	31.43 *
Other	0–4	1606	65.56 *	0.195	0.251	6.52 *	33.38	108.02 *	28.77
	4–8	277	73.55 *	0.208	0.379	6.63 *	39.41	118.76 *	30.11
	8–20	152	78.29 *	0.204	0.502	7.09 *	40.54	122.51 *	31.83
	>20	16	82.68 *	0.199	0.458	8.01 *	38.86	123.74 *	34.09
Total	0–4	562	57.53 *	0.188 *	0.146 *	6.36 *	30.37 *	102.31 *	26.30 *
	4–8	259	66.47 *	0.193 *	0.230 *	6.66 *	32.59 *	107.50 *	28.20 *
	8–20	446	68.04 *	0.199 *	0.283 *	6.57 *	33.33 *	107.50 *	29.92 *
	>20	784	75.25 *	0.204 *	0.413 *	6.73 *	39.47 *	119.50 *	31.24 *

* indicates that the value of different trace elements increased as the number of the different industries increased, which shows the quantitative impacts of industries on trace elements.

**Table 3:** Average concentrations of trace elements in soils from different parent materials (mg/kg).

Soil Parental Materials Type		Sample Quantity	Cr	Pb	Cd	Hg	As	Cu	Zn	Ni
Flooding parental material	Flood alluvial face	102	51.84	50.38 *	0.212 *	0.213	4.86	29.25	111.35 *	23.58
	Plain fluvial face	23	67.73 *	35.07	0.190	0.236	6.37	42.25 *	120.90 *	29.70 *
Estuary alluvial sediment	Modern estuary face	149	61.79	34.00	0.199	0.210	6.21	31.19	90.98	28.23
Stumpy parental material	Granite type residual slope face	25	30.36	46.67 *	0.205	0.108	3.77	20.64	99.54	12.09
	Quaternary Pleistocene Laterite face	6	62.30	48.40 *	0.475 *	0.171	3.67	28.50	95.32	13.75
	Rhyolitic, tuffaceous residual slope face	586	59.41	43.93 *	0.185	0.194	6.64 *	31.86	109.53	26.48
	Purple sandstone residual slope face	9	46.80	31.54	0.112	0.116	4.82	25.53	88.18	17.26
Lacustrine parent material	Lagoon face	655	77.10 *	51.15 *	0.206 *	0.496 *	6.40	38.99 *	118.80 *	30.60 *
Coastal deposition parental material	Silica sand face	168	63.44	28.64	0.196	0.136	6.27	30.73	94.28	29.20
	Aleurite and silt face	293	76.58 *	35.19	0.194	0.209	7.97 *	38.04 *	114.21 *	35.12 *
	Silt face	35	82.07 *	37.39	0.188	0.145	8.76 *	36.67	117.93 *	38.02 *

* indicates a significant level of different trace elements from different soil parental materials.

**Table 4:** Average concentrations of trace elements in the different soil types (mg/kg).

Soil Types	Sample Quantity	Cr	Cd	Hg	As	Cu	Zn	Ni
Coastal saline soil	118	70.40 *	0.168	0.100	8.71 *	36.39 *	104.53	34.39 *
Fluvo-aquic soil	370	67.07	0.195	0.155	6.83 *	32.98	97.36	31.20 *
Skeletal soil	155	60.31	0.186	0.458 *	5.74	34.22	109.54	24.08
Red soil	278	54.68	0.201 *	0.254	5.39	31.72	107.54	22.92
Yellow soil	8	57.71	0.188	0.206	5.17	17.18	90.86	16.07
Paddy soil	1099	72.08 *	0.203 *	0.341 *	6.72 *	36.32 *	117.30 *	30.42 *
Purple soil	5	61.26	0.121	0.117	6.09	27.44	87.14	22.53
Other	18	68.72 *	0.159	0.212	6.18	28.13	92.38	30.18 *

* indicates a significant level of different trace elements in different soil types.

**Table 5:** Values of the SPI for different trace elements.

Element	$P_{i}$ ≤ 1		1 < $P_{i}$ ≤ 2		2 < $P_{i}$ ≤ 3		$P_{i}$ > 3	
	Sample Number	Proportion	Sample Number	Proportion	Sample Number	Proportion	Sample Number	Proportion
Cr	2036	99.27%	14	0.68%	1	0.05%	0	0%
Cd	1917	93.47%	124	6.05%	7	0.34%	3	0.15%
Hg	1421	69.28%	389	18.97%	124	6.05%	117	5.70%
As	2051	100%	0	0%	0	0%	0	0%
Cu	1975	96.29%	73	3.56%	2	0.10%	1	0.05%
Zn	2023	98.63%	25	1.22%	2	0.10%	1	0.05%
Ni	1897	92.49%	137	6.68%	7	0.34%	10	0.49%

**Table 6:** PCA rotation matrix.

Element	Component Matrix			Rotated Component Matrix		
	PC1	PC2	PC3	PC1 (30%)	PC2 (25%)	PC3 (15%)
Cr	0.82	−0.39	0.06	0.88	0.18	0.15
Cd	0.34	0.73	−0.23	−0.20	0.81	0.04
Hg	0.40	0.28	0.53	0.12	0.26	0.67
As	0.57	−0.47	0.03	0.73	−0.01	0.04
Cu	0.81	0.27	−0.14	0.45	0.72	0.12
Zn	0.72	0.44	−0.20	0.28	0.82	0.10
Ni	0.75	−0.46	−0.10	0.87	0.14	−0.03

**Table 7:** FMDM parameters and thresholds.

Elements	Class	Proportion%	Mean (mg/kg)	STD (mg/kg)	Freedom	χ^2^	*p*	Cutoff Value (mg/kg)	Background Values
Cr	2	35.79%	49.51	1231.02	52	51.47	0.15	52.49	68.7
		64.21%	77.54	226.39					
Cd	1	100%	0.18	0.006	45	48.06	0.35	-	0.157
Hg	3	45.35%	0.09	0.001	20	16.27	0.7	0.156	0.11
		41.21%	0.30	0.002				0.566	
		13.44%	0.91	0.12					
As	2	7.28%	2.74	0.75	41	51.81	0.12	2.85	6.23
		92.72%	6.85	6.05					
Cu	1	100%	34.65	206.12	55	59.05	0.33	-	29.2
Zn	1	100%	127.80	3857.11	68	53.54	0.09	-	89.9
Ni	2	38.51%	21.8	290.52	30	28.76	0.53	22.77	26.3
		61.49%	31.29	39.94					

STD: standard deviations of log-normal distribution; χ^2^: Chi-square goodness-of-fit statistics. *p* means that the estimated model is consistent with the observed distribution; when *p* 0.05, *H*_0_ is rejected.
